# Development of Efavirenz nanoparticle for enhanced efficiency of anti-retroviral therapy against HIV and AIDS

**DOI:** 10.1186/1471-2334-12-S1-P7

**Published:** 2012-05-04

**Authors:** BN Vedha Hari, K Dhevendaran, N Narayanan

**Affiliations:** 1 Department of Pharmacy, School of Chemical and Bio-Technology, SASTRA University, Thanjavur-613 401, Tamil Nadu, India; 2 Department of Pharmaceutics, Madras Medical College, Chennai-600001, Tamil Nadu, India

## Background

The FDA approved drug Efavirenz is a Non-Nucleoside Reverse Transcriptase Inhibitor (NNRTI) successful first line drug of choice in Highly Active Anti-Retroviral Therapy (HAART) for treatment of HIV and AIDS. It is poorly water soluble drug (10 g/ml) with 40-45% of bioavailability and administered as high doses 600-800 mg/day. Increase in solubility can enhance bioavailability; providing reduction of dose, resistance and harmful side effects.

## Methods

Efavirenz nanoparticles are developed using methacrylate polymers (Eudragit E100) by emulsion solvent evaporation method (1:0.5, 1:1, 1:2 and 2:1 ratios) and the in-vitro evaluations such as particle size, morphology, solubility changes, drug release, compatibility and cytotoxicity tests are carried out.

## Results

The particle size of 99-200 nm with narrow size distribution and surface charge (-52 V) shows high stability. The formulation with entrapment efficiency (75-90%) shows higher drug release profile 95-100% within 1 hour compared to 23%-58% of pure drug in water, 0.1N HCl and phosphate buffer pH 7.4 media. The DSC, TG-DSC, powder XRD and SEM morphology results reveal that there is solid transition from crystalline structure to amorphous state, which supports the solubility enhancement. The FT-IR gives the compatibility results for drug with other excipients. The Efavirenz nanoparticles subjected for in-vitro cytotoxicity and cell uptake studies using monocytes / macrophages (THP-1) proved better uptake (Flocytometry and Confocal microscope) of nanoparticles than free drug.

## Conclusion

The solubility enhancement due to nanosizing helps in hastening the drug release and also increasing cell uptake, which helps in attaining high bioavailability with low dose of Efavirenz.

